# Let-7 miRNAs are Selectively Sensitive to Dicer Dosage

**DOI:** 10.17912/micropub.biology.002123

**Published:** 2026-04-23

**Authors:** Dylan Rothbort, Sharan Malagobadan, Indranil Mondal, Acong Yang, Shuo Gu

**Affiliations:** 1 RNA Biology Laboratory, National Cancer Institute, Frederick, MD, U.S.

## Abstract

DICER1 syndrome is a rare cancer predisposition disorder linked to disruption of the miRNA biogenesis enzyme Dicer. To model germline DICER1 loss, we generated heterozygous knockout cell lines in mouse and human systems. While global miRNA levels were largely unaffected, the let-7 family was consistently downregulated across all models. The coordinated reduction of both guide and passenger strands, without similar effects on co-transcribed miRNAs, indicates a post-transcriptional biogenesis mechanism. These findings reveal a selective sensitivity of let-7 miRNAs to Dicer dosage and suggest a role for let-7 dysregulation in DICER1-associated tumorigenesis.

**Figure 1. Evaluation of miRNA expression in mouse and human DICER1 HetKO cells:** **a** ) Volcano plot showing differential miRNA expression in P19 cells (HetKO/WT). Each dot represents a miRNA colored by its WT expression level (log₁₀ RPM). Dashed lines indicate significance thresholds (adjusted P < 0.05, fold change < 0.75). **b** ) Boxplot of GYM cleavage score for significantly downregulated, unchanged, and upregulated miRNAs in P19 cells. GYM scores were calculated as previously described (Lee et al., 2023). Only mouse miRNAs with calculable GYM scores are shown. A two-sided Mann-Whitney U test was used to calculate P values; ns, not significant. Boxplots show the median (center line), 25th–75th percentile (box), and extreme values within 1.5x the interquartile range (whiskers). **c** ) Venn diagram showing overlap of downregulated miRNAs (fold change <0.75) between mouse P19 cells and human HCT116, and HEK293T cells. Numbers indicate miRNAs unique to or shared among datasets. The four mutually downregulated miRNAs are listed in red. **d-f** ) MA plots showing differential miRNA expression in P19, HCT116 and HEK293T cells, respectively. Log _2_ fold change (HetKO/WT) is plotted against average expression (log10) using spike-in normalized reads. miRNAs from the let-7 cluster are labeled as let-7 guide strands (blue), let-7 passenger strands (red), or non–let-7 miRNAs (orange). **g–i)** Boxplots summarizing log2 fold change (HetKO/WT) for let-7 clustered miRNAs in P19, HCT116 and HEK293T cells, corresponding to panels d-f. Numbers of miRNAs in each category are indicated. A two-sided Mann-Whitney U test was used to calculate P values; ns, not significant. Boxplots show the median (center line), 25th–75th percentile (box), and extreme values within 1.5x the interquartile range (whiskers).

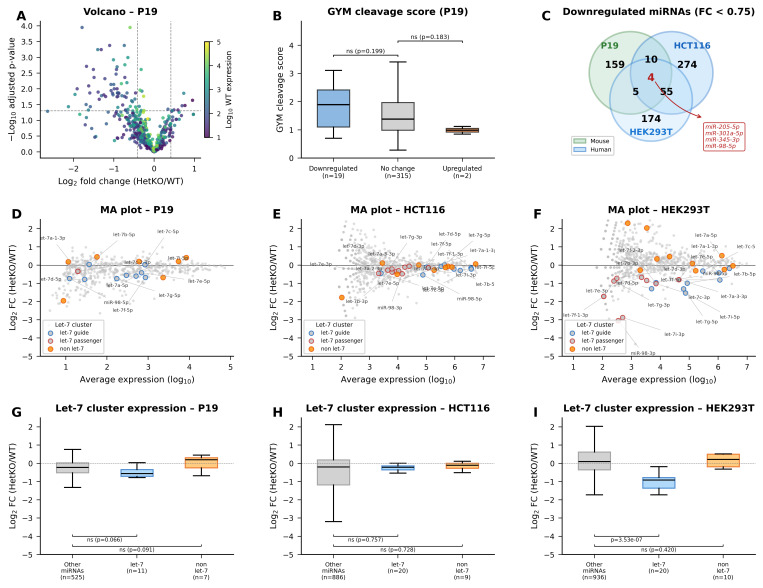



## Description

MicroRNAs (miRNAs) are a key class of small noncoding RNAs involved in post-transcriptional regulation (Bartel, 2018; Kim et al., 2025). Canonical miRNA biogenesis begins with the transcription of primary miRNAs (pri-miRNAs). These pri-miRNAs are processed in the nucleus by the RNase III endonuclease Drosha, in complex with its cofactor, the RNA-binding protein DiGeorge Syndrome Critical Region 8 (DGCR8), to generate precursor miRNAs (pre-miRNAs). These pre-miRNAs are then exported to the cytoplasm by exportin 5 (XPO5), where they are further processed by Dicer (encoded by *DICER1* ), the cytoplasmic counterpart of Drosha, to produce mature miRNAs.

Heterozygous loss-of-function (LOF) of *DICER1* underlies the autosomal dominant *DICER1* syndrome, a cancer predisposition disorder (de Kock et al., 2019; Foulkes et al., 2014; Hill et al., 2009). Tumor development in this setting does not follow a classical two-hit model. Instead, it typically involves a germline LOF mutation in one *DICER1* allele followed by acquisition of a somatic hotspot mutation which specifically abolishes the cleavage activity of the RNase IIIb domain (Heravi-Moussavi et al., 2012). Although these hotspot mutations have been extensively characterized and are now understood to exhibit both loss- and gain-of-function properties (Jee et al., 2025; Malagobadan et al., 2025), the contribution of the germline LOF mutation, namely the loss of a single *DICER1* allele, remains less well defined.

Mouse models have shown that *DICER1* haploinsufficiency increases tumor susceptibility, yet whether this effect is mediated through perturbation of miRNA biogenesis remains unclear (Kumar et al., 2009). Notably, prior work, including ours, suggests that heterozygous loss of *DICER1* has minimal global impact on miRNA processing (Malagobadan et al., 2025). These observations raise the possibility that more subtle or selective effects on miRNA biogenesis may underlie the pathogenic consequences of *DICER1* haploinsufficiency. To address this, we characterized *DICER1* heterozygous knockout (HetKO) cell lines.

We first profiled miRNA expression in HetKO mouse embryonic carcinoma (P19) cells. Consistent with prior observations, global miRNA abundance was largely preserved upon loss of a single Dicer allele. Nonetheless, a subset of miRNAs showed modest changes, with more species decreased than increased in abundance ( **Figure 1A** ). These effects were largely confined to lowly expressed miRNAs, indicating no broad impairment of miRNA biogenesis. We next asked whether reduced Dicer levels preferentially affect suboptimal substrates. Dicer cleavage depends on intrinsic features of pre-miRNAs, including the GYM motif (Lee et al., 2023). However, GYM cleavage scores did not differ significantly between downregulated and upregulated miRNAs ( **Figure 1B** ), arguing against differential processing efficiency as the primary driver. To assess generality, we generated *DICER1* HetKO lines in human HEK293T and HCT116 cells **.** Most miRNAs reduced in HetKO P19 cells were largely unchanged in these systems ( **Figure 1C )** , indicating Dicer haploinsufficiency does not produce broad or consistent alterations in miRNA expression across cell types.

Interestingly, miR-98-5p, a member of the let-7 family, was among the few miRNAs consistently reduced across both human and mouse *DICER1* HetKO cell lines. This observation prompted us to examine whether this effect extended to other let-7 family members. We therefore analyzed all expressed let-7 miRNAs across the three HetKO models ( **Figure 1D- F** ). Strikingly, although the magnitude of change was modest for most members (median log2 fold change = -0.433), the let-7 family showed a consistent trend toward reduced expression, with no members exhibiting increased abundance. This pattern was observed across both mouse and human systems, suggesting a coordinated and non-random effect on this miRNA family.

To gain insight into the underlying mechanism, we first examined both strands of let-7 miRNAs. Notably, both guide and passenger strands were consistently reduced across human and mouse HetKO datasets. Because only the guide strand is incorporated into mature RISC, the coordinated decrease of both arms argues against mechanisms acting at the post-RISC stage, including target-mediated effects such as target-mediated miRNA decay, and instead points to regulation at the level of miRNA biogenesis. We next asked whether this effect could reflect altered transcription. Many let-7 miRNAs are co-transcribed with non-let-7 miRNAs within the same pri-miRNA clusters, allowing the latter to serve as internal controls. However, these co-expressed non-let-7 miRNAs did not show similar changes ( **Figure 1G- I** ), indicating that transcriptional differences are unlikely to account for the observed reduction in let-7 expression.

Overall, our findings indicate that heterozygous loss of *DICER1* does not broadly impair miRNA biogenesis but instead leads to a subtle yet consistent downregulation of the let-7 family. These results suggest that let-7 miRNAs may be particularly sensitive to Dicer dosage or subject to specific regulation during their biogenesis. However, the mechanistic basis by which reduced Dicer levels selectively impact let-7 expression remains to be elucidated. Previous studies have shown that the let-7 miRNAs can directly target Dicer expression (Forman et al., 2008; Jakymiw et al., 2010; Xie et al., 2003), raising the possibility of a feedback relationship. In this context, reduced Dicer levels may lead to decreased let-7 expression, thereby relieving repression of Dicer and partially buffering the reduction on Dicer level, potentially contributing to homeostatic control.

A limitation of this study is that we quantified only mature miRNA levels by miR-seq and did not measure the corresponding precursor (pre-let-7), the direct substrate of Dicer. Standard small RNA sequencing libraries primarily capture mature miRNAs, and pre-miRNAs are generally not represented, limiting our ability to assess precursor abundance from these data. If reduced mature let-7 levels result from impaired Dicer processing, one might predict accumulation of pre-let-7. However, pre-let-7 is subject to TUT4/7-mediated uridylation and subsequent DIS3L2-dependent degradation (Chang et al., 2013; Heo et al., 2009), which can limit its steady-state accumulation. Thus, reduced processing efficiency may not necessarily lead to detectable increases in pre-let-7 levels. Our data therefore support the dose sensitivity of let-7 to Dicer and point to a defect at a post-transcriptional stage during miRNA biogenesis, but do not resolve the precise mechanistic step affected. Future studies directly quantifying pre-let-7, together with in vitro Dicer cleavage assays, will be important to distinguish between altered processing efficiency and changes in precursor stability.

Given the well-established tumor-suppressive roles of let-7 family members, their coordinated downregulation provides a plausible mechanistic link between *DICER1* haploinsufficiency and increased tumor susceptibility. We propose that selective reduction of let-7 miRNAs may represent one contributing factor underlying the pathogenic consequences of *DICER1* germline loss.

## Methods

Small RNA libraries for miRNA-seq were generated from 500ng total RNA using the QIAseq miRNA Library Kit (Qiagen, cat # 331502) according to the manufacturer’s instructions. For normalization, 1 μl of QIAseq miRNA Library QC Spike-ins (Qiagen, cat # 331535) was added to each sample before library preparation. Libraries were size-selected on a native 6% (w/v) acrylamide gel, purified using ethanol precipitation, and sequenced on an Illumina NextSeq 1000 platform. miRNA abundance was calculated using QuagmiR and normalized to spike-in controls (Bofill-De Ros et al., 2019). GYM cleavage scores were obtained from Lee et al. (2023) and mapped to the corresponding mouse orthologs. Python code was used to generate panels with assistance from an AI tool (Claude Opus 4.6, Anthropic), with the plots reviewed and validated by the authors.
